# Development of Validated Methods and Quantification of Curcuminoids and Curcumin Metabolites and Their Pharmacokinetic Study of Oral Administration of Complete Natural Turmeric Formulation (Cureit™) in Human Plasma via UPLC/ESI-Q-TOF-MS Spectrometry

**DOI:** 10.3390/molecules23102415

**Published:** 2018-09-20

**Authors:** Shintu Jude, Augustine Amalraj, Ajaikumar B. Kunnumakkara, Chandradhara Divya, Bernd-Michael Löffler, Sreeraj Gopi

**Affiliations:** 1 R&D Centre, Aurea Biolabs (P) Ltd., Kolenchery, Cochin-Kerala 682311, India; shintu.jude@plantlipids.com (S.J.); amalraj.a@plantlipids.com (A.A.); 2 Department of Biosciences and Bioengineering, Indian Institute of Technology Guwahati, Guwahati, Assam-781039, India; kunnumakkara@iitg.ernet.in; 3 #2/5, Dahlia Building, 3rd Floor, 80 Feet Road, RMV 2nd Stage, Bengaluru, Karnataka 560094, India; divya@bioagiletherapeutics.com; 4 Institute of Mitochondrial Medicine, Pfalzburger Str. 43/44, 10717 Berlin, Germany; loeffler@imm.institute

**Keywords:** curcuminoids, curcumin metabolites, UPLC/ESI-Q-TOF-MS spectrometry, pharmacokinetic, bioavailable curcumin (Cureit™)

## Abstract

Specific and sensitive ultra-high performance liquid chromatography-quadrupole time of flight-mass spectroscopy (UPLC-QTOF-MS) methods have been developed for the determination of curcuminoids and curcumin metabolites in human blood plasma. The UPLC-QTOF-MS method used a binary solvent delivery system and the chromatographic separation was performed on a C-18 (2.1 × 50 mm; 1.7 µm) column. Mass spectra were obtained on a Waters Xevo G2S Q-TOF mass spectrometer. The developed methods to characterize the pharmacokinetics of curcuminoids and curcumin metabolites in human blood plasma after an oral administration of bioavailable curcumin—Cureit™—were validated. It was found that the complete turmeric matrix enhances the concentration of tetrahydrocurcumin (THC), hexahydrocurcumin (HHC), octahydrocurcumin (OHC), curcumin-*O*-glucuronide (COG) and curcumin-*O*-sulfate (COS) in the blood plasma once the product is administrated.

## 1. Introduction

Curcuminoids, a combination of curcumin (CUR), demethoxycurcumin (DMC) and bisdemethoxycurcumin (BDMC) are natural polyphenols extracted from the rhizome of turmeric (*Curcuma longa*) [1]. CUR is the main active constituent of turmeric and is extensively utilized as preservative, flavoring and coloring agent in beverages and in foods [2]. Curcuminoids from turmeric have been shown to have a large series of biological and pharmacological activities including antioxidant, anti-inflammatory, antimicrobial, anticancer, radio-protective, cardio-protective, neuroprotective and antitumor effects, the majority of which have been demonstrated by various preclinical studies [3,4]. The plasma levels of curcuminoids are extremely low (<50 ng/mL), even after an oral intake of curcuminoids up to 12 g/day [1,5]. This low oral bioavailability of curcuminoids is considered due to their poor solubility, unsatisfactory absorption, and rapid metabolism [1,6]. Because of the low oral bioavailability, it is very tough to assess the absorption of curcuminoids by monitoring their original forms. Metabolic studies have established that orally ingested CUR is comprehensively altered via conjugation and reduction in both rodents and human [1,7]. Many analytical methods were developed using thin layer chromatography (TLC), capillary electrophoresis (CE), gas chromatography-mass spectrometry (GC-MS), high performance liquid chromatography (HPLC) and its coupling to mass spectrometry (LC-MS) for the analysis of chemical content of various turmeric samples and curcuminoid formulations, as well as, for tracking the CUR metabolism in the in vitro and in vivo studies. These studies demonstrated that CUR undergoes extensive metabolism through conjugation and reduction [8,9,10]. Early studies suggested that, following oral administration, CUR undergoes metabolic-*O*-conjugation to curcumin-*O*-glucuronide (COG) and curcumin-*O*-sulfate (COS) and bioreduction to tetrahydrocurcumin (THC), hexahydrocurcumin (HHC) and octahydrocurcumin (OHC) in rats and mice in vivo [10,11,12,13], in suspensions of human and rat hepatocytes as well as in human and rat intestinal cytosol [10,14]. But, to the best of our knowledge there are no perceptive bioanalytical methods reported for the estimation of CUR, DMC and BDMC and CUR metabolites in a picogram level in human blood plasma. Ultra-performance liquid chromatography coupled with quadrupole time-of-flight mass spectrometry (UPLC/ESI-Q-TOF-MS) is a high-resolution selective and sensitive technique that can be used to investigate and recognize metabolites with mass accuracy.

**Figure 1:**
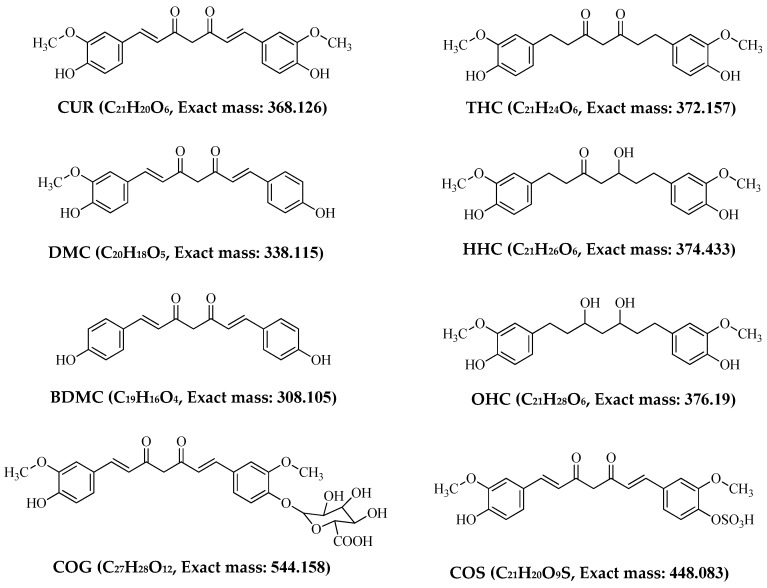
Chemical structures and theoretical masses of curcuminoids and curcumin metabolites.

In this study, we developed three new mass spectrometry methods for quantification of: (i) CUR, DMC, BDMC and COG, (ii) THC, HHC and OHC and (iii) COG using UPLC connected to a Waters Xevo G2-S-TOF instrument under negative ion mode. The chemical structure of the curcuminoids nd curcumin metabolites are given in Figure 1.

The experiments should ultimately support drug design of future chemo preventives derived from CUR. The optimized UPLC-Q-TOF-MS method was validated by using human plasma. Its capability to measure the curcuminoids and curcumin metabolites in human plasma was confirmed by analyzing the plasma samples from healthy human subjects after an oral administration of Cureit™, a bioavailable form of curcumin. The method was applied to identify and quantify the levels of parent curcuminoids and CUR metabolites in biometrics of human after administration of Cureit™. The Cureit™ was prepared by using polar-nonpolar-sandwich technology with complete natural turmeric matrix [15]. The extensive studies on the therapeutic properties exhibited by Cureit™ demonstrated its benefits such as anticancer activity [16], elastase inhibition activity [17], antioxidant activity [18] and anti-aging activity [19] with a proven higher bioavailability [20]. Additionally, detection of chemical distinctiveness of the curcuminoids and CUR metabolites was confirmed by mass spectrometry. The present technique showed excellent performance for the bioanalysis of number of samples with respect to its high sensitivity and very low retention time.

## 2. Results and Discussion

### 2.1. Mass Spectrometric Analysis of CUR, DMC, BDMC, COG, COS, THC, HHC and OHC

The mass spectra of CUR, DMC, BDMC, COG, COS, THC, HHC and OHC were acquired by the mass spectrometer coupled with an electrospray ion source under both positive and negative ion modes. The signal intensities of the studied ions CUR, DMC, BDMC, COG, COS, THC, HHC and OHC were higher in negative ion mode than the positive ion mode (Table 1) due to the existence of ketone group in the curcuminoids and the CUR metabolites [21]. Negative mode was selected for the analysis, as the signal strengths were higher in negative mode for the standard solutions under direct infusion, as shown in the Table 1. Accordingly, in this study, the mass spectra were acquired under a negative ion mode only.

**Figure 2:**
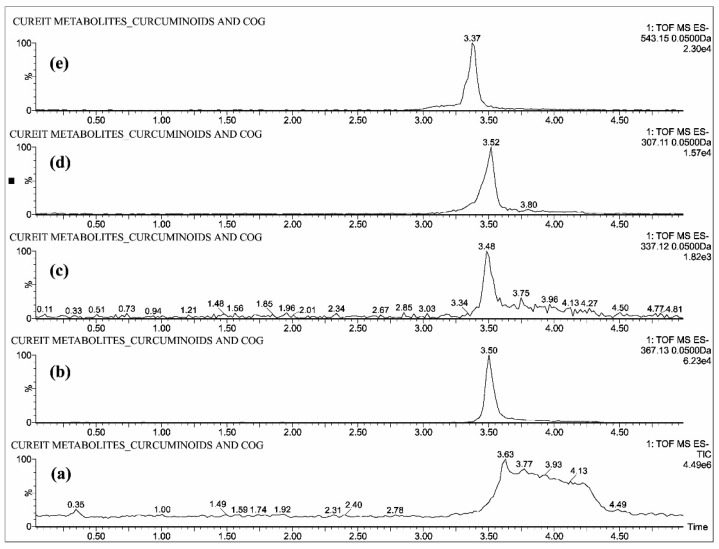
UPLC-QTOF-MS chromatograms of standards—(**a**) total ion chromatogram of curcuminoids and COG, extracted ion chromatograms of (**b**) CUR, (**c**) DMC, (**d**) BDMC and (**e**) COG.

**Figure 3:**
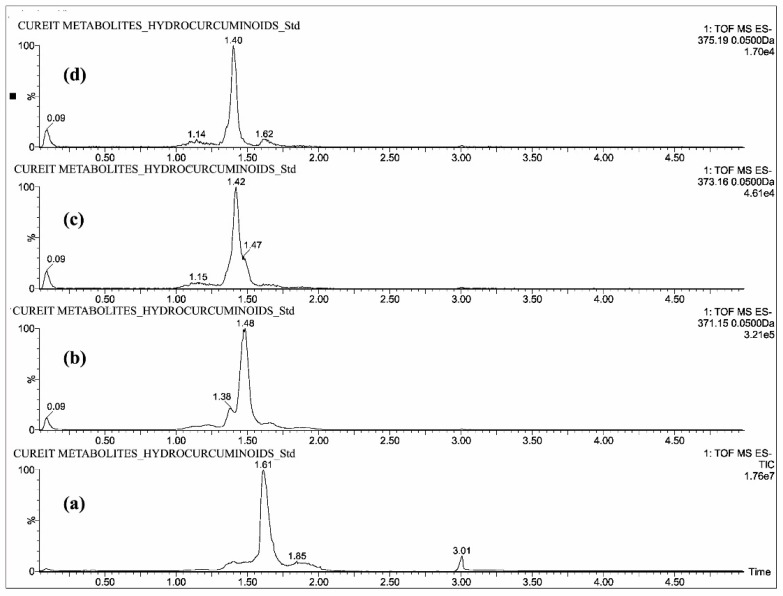
UPLC-QTOF-MS chromatograms of standards—(**a**) total ion chromatogram of hydrogenated curcuminoids, extracted ion chromatograms of (**b**) THC, (**c**) HHC and (**d**) OHC.

**Figure 4:**
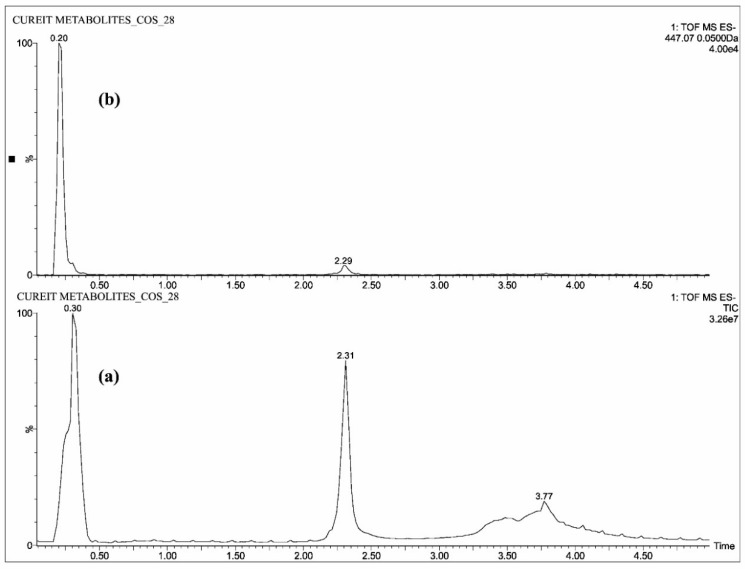
UPLC-QTOF-MS chromatograms of standard—(**a**) total ion chromatogram of COS and (**b**) extracted ion chromatogram of COS.

The individual solutions were prepared by mixing 10 µg/mL of CUR, DMC, BDMC, COS and COG in methanol; 10 µg/mL of THC, HHC and OHC were prepared with mixture of 50% methanol/water, as the direct infusion of hydrocurcuminoids to TOF gave a maximum intensity while dissolving in the same. The full scan mass spectra showed predominant deprotonated molecular ions [M − H]^−^ of *m*/*z* 367.13, 337.12, 307.11, 543.15, 447.07, 371.15, 373.16 and 375.19 for CUR, DMC, BDMC, COG, COS, THC, HHC and OHC respectively (Figure 2, Figure 3 and Figure 4).

Optimum chromatographic separation of CUR, DMC, BDMC and COG was attained by using acetonitrile and methanol (70:30) with 10 mM ammonium formate in water, with a flow rate of 400 µL/min. In the case of THC, HHC and OHC, the best separation was achieved by acetonitrile with 0.1% ammonium acetate in water, with a flow rate of 500 µL/min. Analysis of COS was conducted with 0.1% ammonia and acetonitrile with a flow rate of 400 µL/min. 

**Figure 5:**
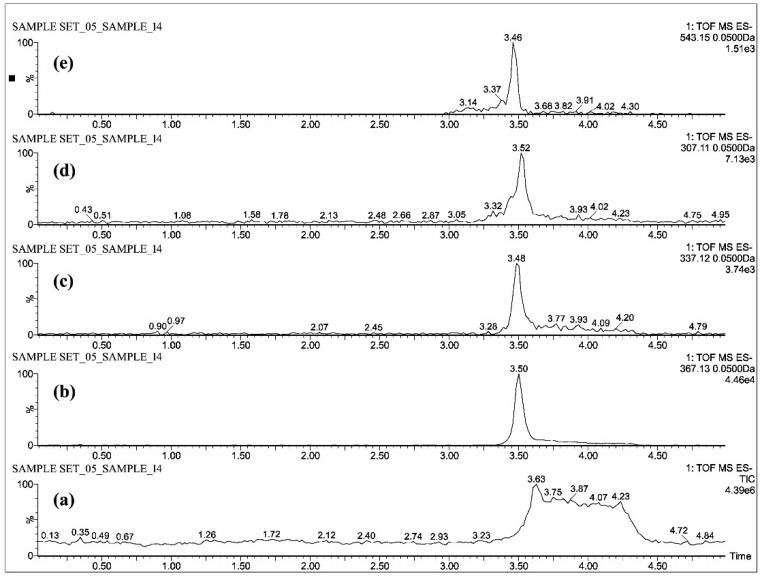
UPLC-QTOF-MS chromatograms of a plasma sample—(**a**) total ion chromatogram of curcuminoids and COG, extracted ion chromatograms of (**b**) CUR, (**c**) DMC, (**d**) BDMC and (**e**) COG.

**Figure 6:**
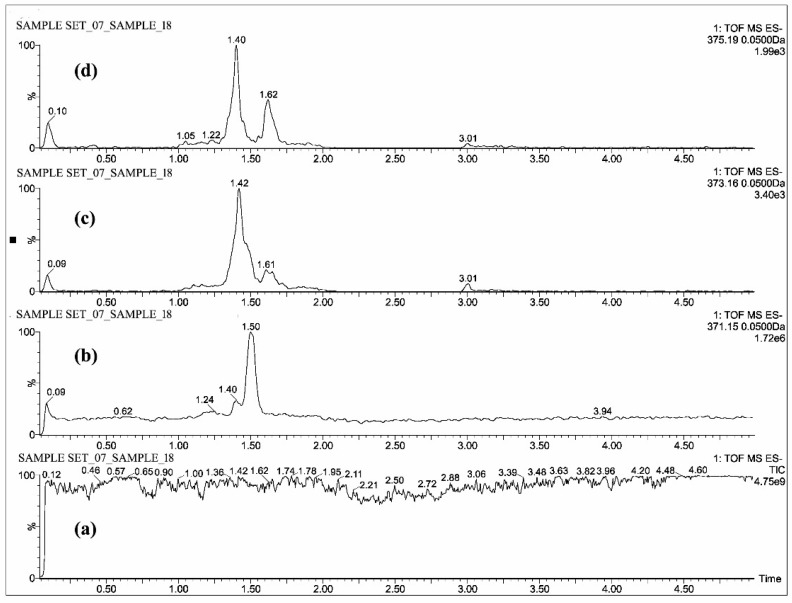
UPLC-QTOF-MS chromatograms of a plasma sample—(**a**) total ion chromatogram of hydrogenated curcuminoids, extracted ion chromatograms of (**b**) THC, (**c**) HHC and (**d**) OHC.

**Figure 7:**
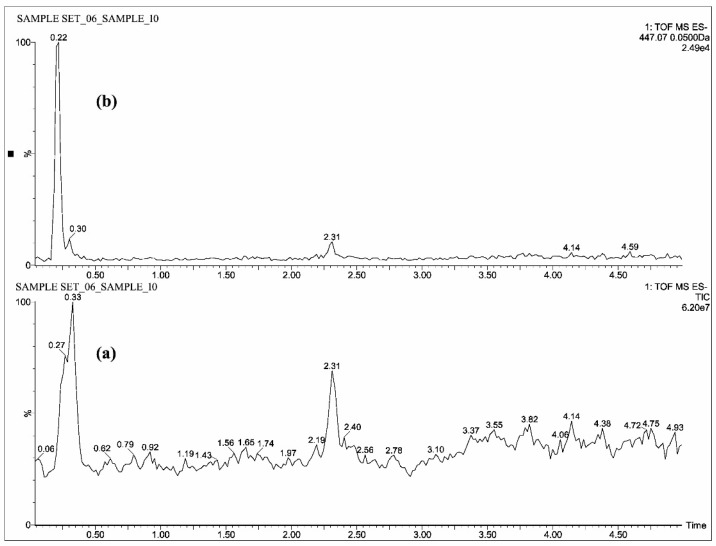
UPLC-QTOF-MS chromatograms of a plasma sample—(**a**) total ion chromatogram of COS and (**b**) extracted ion chromatogram of COS.

All the blood plasma samples were analyzed and the resultant chromatograms showed retention times at 3.50, 3.48, 3.52 and 3.46 min for CUR, DMC, BDMC and COG respectively (Figure 5) and the resulting chromatograms for THC, HHC and OHC showed the retention times at 1.48, 1.42 and 1.4 min respectively (Figure 6). For COS, the chromatogram showed the retention time at 0.22 min (Figure 7). This is the distinctive study for the determination of CUR, DMC, BDMC and COG in a single method, as well as hydrogenated curcumin such as THC, HHC and OHC are quantified in another single method. Furthermore, COS is determined in a separate method.

### 2.2. Method Validation

The method was established to be precise through extracted blank plasma when compared with plasma samples spiked with CUR, DMC, BDMC, COG, COS, THC, HHC and OHC standards, which did not show any interference at the respective retention time of each standard. Under optimal QTOF-MS conditions, the calibration curves of CUR, DMC, BDMC, COG, COS, THC, HHC and OHC showed linearity over the concentration range of 1.0 to 1000 ng/mL with linear regression coefficients greater than 0.998. The intra-day and inter-day accuracy and reproducibility of CUR, DMC, BDMC, COG, COS, THC, HHC and OHC in human blood plasma were evaluated at concentrations of 4, 8, 16, 32, 64, 128 and 256 ng/mL. The results are summarized in Table 2 and Table 3. The intra-day and inter-day LLOQs at 1.0 ng/mL for CUR, DMC, BDMC, COG, COS, THC, HHC and OHC were with a coefficient of variation lesser than 20% and signal to noise ratio greater than 5. The intra assay and inter assay accuracy in terms of % bias and precision in terms of % relative standard deviation were given in Table 2 and Table 3, respectively. All the quality controls for the intra-day and inter-day validation showed coefficient of variations of less than 15% and accuracy between 85% and 115%. This is confirmed that the current methods have satisfactory accuracy, precision and reproducibility for the simultaneous quantification of all analytes throughout a wide range of concentrations. No carry-over peaks were observed at the corresponding retention times when blank mobile phase samples were injected after standard samples. The developed methods have shown high degree of accuracy, sensitivity, reproducibility, simplicity and also provide short analysis time (5 min).

### 2.3. Pharmacokinetic Study of Orally Administrated Bioavailable Form of Curcumin

**Figure 8:**
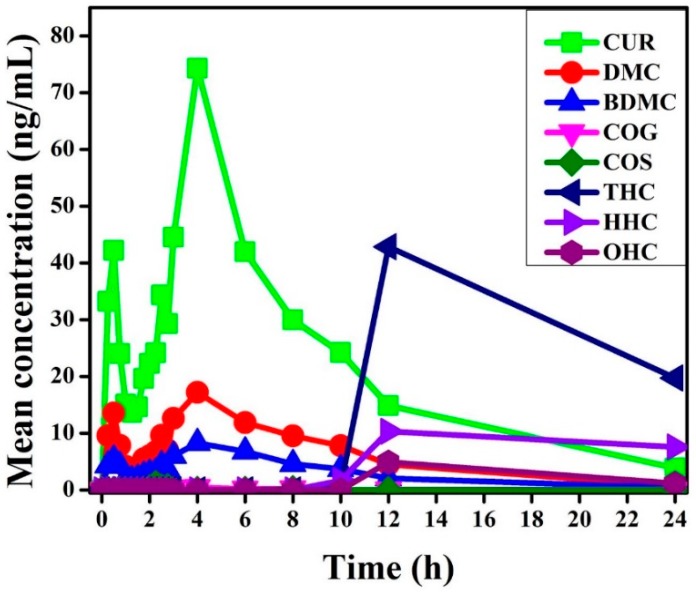
Pharmacokinetic profiles of CUR, DMC, BDMC, COG, COS, THC, HHC and OHC concentration in plasma at different time intervals after administration of a single dose of Cureit™ (500 mg/day).

The above described methods were applied to measure the levels of CUR, DMC, BDMC, COG, COS, THC, HHC and OHC in plasma samples collected from 15 healthy participants (Figure 8) and the pharmacokinetic parameters in plasma obtained from the collected concentration-time data are summarized in Table 4. After an oral administration of Cureit™—a bioavailable form of curcumin (500 mg)—the level of CUR in the plasma initially showed a rapid increase and reached a C_max_ of 74.31 ng/mL at 4 h and was remained detectable up to 24 h. DMC and BDMC were reached their C_max_ of 17.19 ng/mL and 8.28 ng/mL respectively at 4 h. COG was detected C_max_ of 0.862 ng/mL at 2.5 h. The detection of THC was started at 10 h and reached C_max_ of 42.84 ng/mL at 12 h. In the case of HHC, a C_max_ of 10.33 ng/mL was detected at 12 h and OHC has shown a C_max_ of 4.91 ng/mL at 12 h. COS was initially detected at the 1.75 h and showed a C_max_ of 1.06 ng/mL at 2.25 h.

Curcuminoids is a mixture of CUR, DMC and BDMC. Even though the pharmacokinetic study of CUR has been studied extensively, the same of the DMC and BDMC remains unexplored owing to their relatively low abundance in the viable form of curcuminoids [22,23]. Curcuminoids have also shown their reduced metabolites of curcuminoids such as THC and HHC and two other major conjugates are COG and COS [23,24,25]. One of our previous study has demonstrated the improved bioavailability of curcuminoids by the oral administration of Cureit™ against 95% curcuminoids [20]. Another study compared the oral bioavailability of Cureit™ with two other commercially available formulations in healthy human male adult and demonstrated that Cureit™ with the complete natural turmeric matrix formulated by the polar non-polar sandwich (PNS) technology [15] exhibited greater bioavailability than the other formulations [26]. The pharmacokinetic study results are also supported that Cureit™ can enhance the absorption of CUR by the registration of highest AUC and C_max_ values for CUR (Table 4). Furthermore, this study simultaneously characterized and quantitatively determined the metabolites of CUR such as THC, HHC, OHC, COG and COS along with CUR, DMC and BDMC in human plasma after an oral administration of 500 mg Cureit™. As shown in Figure 8; COG, COS, THC, HHC and OHC were detected in human blood plasma 1, 1.75, 10, 10 and 12 h after oral administration of 500 mg of Cureit™, respectively. Based on the plasma concentration-time profile of curcuminoids and CUR metabolites, CUR is the predominant circulating compound, followed by THC, DMC, HHC, BDMC, OHC, COS and COG (Figure 8).

A responsive, discriminatory and quick UPLC/QTOF-MS method for the quantification of CUR, DMC and BDMC was developed by Ahmad et al. [21] and successfully employed in Wistar rodent brain homogenate with acceptable precision and satisfactory accuracy. Cao et al. [1] reported a liquid chromatography/tandem mass spectrometry (LC-MS/MS) method to quantify CUR and its metabolites simultaneously in human plasma and studied the pharmacokinetic of curcumin and its metabolites using a curcumin nanoemulsion. Verma et al. [27] also developed a validated UPLC-QTOF-MS method for the determination of curcuminoids and their pharmacokinetic study in Swiss mice. Lou et al. demonstrated the gastrointestinal metabolism of CUR in the presence of human intestinal microflora using QTOF-MS. The study detected and identified a total twenty three curcumin metabolites by in vitro [10]. Among them COG and COS and THC are well known curcumin metabolites [1]. The above studies demonstrated the quantification and pharmacokinetic studies of curcumin and its metabolites in various methods and by using different instruments. Among these studies, very few were detected hydrogenated curcumin, particularly THC and HHC in animal and human plasma; more over there were no studies for identification or quantification of OHC in the blood plasma. In the present study, three hydrogenated curcumin; THC, HHC and OHC were detected and quantified by the newly developed methods.

In this study, three validated methods were developed, which are very feasible in terms of accuracy, precision, sensitivity, specificity and stability of the analytes. The method was utilized for the determination of CUR, DMC, BDMC and COG either individually or simultaneously in a single method, hydrogenated curcumin such as THC, HHC and OHC were also analyzed alone or concurrently in another single method in human plasma. COG was analyzed individually in the human plasma.

## 3. Materials and Methods

### 3.1. Chemicals and Reagents

Standards of curcumin (≥97%), demethoxycurcumin (≥94%), bisdemethoxycurcumin (≥94%), tetrahydrocurcumin (≥96%) and hexahydrocurcumin (≥90%) were purchased from Sigma Aldrich, Mumbai, India. The internal standard (IS), hesperetin was purchased from Sigma Aldrich, Steinheim. Octahydrocurcumin (≥92%) was prepared in-house and characterized by mass spectroscopy and melting point analysis. Curcumin-O-glucuronide (≥98%) and curcumin-O-sulfate (≥98%) were purchased from Clearsynth laboratories, Mumbai, India. The complete natural turmeric matrix formulation, Cureit™ (bioavailable form of curcumin) was obtained from Aurea Biolabs Pvt. Ltd., Cochin, India. Leucine enkephalin and sodium formate were purchased from Waters, Milford, MA, USA. All other solvents and chemicals were of LC-MS grade and were purchased from J.T. Baker, Center Valley, PA, USA. Millipore-MilliQ distilled water was utilized during the complete study.

### 3.2. Instrumentation

A Waters ACQUITY UPLC I-class system (Waters, Milford, MA, USA) equipped by a binary solvent delivery system was used and the chromatographic separation was executed on a ACQUITY UPLC BEH C-18 (2.1 × 50 mm; 1.7 µm) column. Mass spectra were recorded by a Xevo G2S Q-TOF (Waters, Milfor, MA, USA) mass spectrometer. The mass spectrometer with electrospray ionization (ESI) was used for the MS analysis in the negative mode, and was connected to the MassLynx V4.1 (http://www.waters.com/waters/supportList.htm?cid=511442&q=MassLynx%2520release%2520notes&qTemp=MassLynx+release+notes) software. Negative mode was selected for the analysis, as the signal strengths were higher in negative mode for the standard solutions under direct infusion. Lock mass correction was applied using 2 ng/µL Leucine-enkephalin solution in 50:50 (*v*/*v*) acetonitrile: water containing 0.1% formic acid. It was infused with 10 µL/min flow rate and the mass-to-charge ratio (*m*/*z*) 554.252 of Leucine-enkephalin was set to be monitored in an interval of 10 s with a scan time 0.3 s. The mass window set for the lockmass was ±0.3 Da.

The levels of CUR, DMC, BDMC and COG were monitored by the following ESI parameters such as capillary voltage 2.42 kV, sample cone voltage 42 V with source temperature 140 °C and desolvation temperature 600 °C. The cone gas flow was maintained as 30 L/h and desolvation gas flow was 800 L/h. Data were collected in continuum mode. A binary solvent system was set with solvent A containing 70:30 acetonitrile: methanol and solvent B containing 10 mM ammonium formate in water. The flow of 0.4 mL was maintained with an initial composition of solvent A: B as 5:95 up to 1.2 min, then the composition was changed to 95:5 up to 3.7 min. Then the composition was modified to 5:95 up to end of the 5 min run time.

The concentrations of THC, HHC and OHC were screened with slightly modified earlier ESI parameters viz. capillary voltage 3 kV, sample cone 40 V, source temperature and desolvation temperature were kept as 140 °C and 250 °C respectively. The cone gas flow was maintained at 50 L/h and the desolvation gas flow at 800 L/h. Here also, the data were collected in continuum mode. In the solvent system, solvent A was 10 mM ammonium acetate in water and solvent B was acetonitrile. The flow was maintained as 0.5 mL with an initial composition of A: B at 90:10 ratio up to 1 min and the composition was changed to the ratio 2:98 up to 3.5 min. Again, the composition was restored as 90:10 ratio up to the end of 5 min run time.

For COS screening, the ESI parameters and gas flow rates were similar to curcuminoids. Data collected in continuum mode. The binary solvent system consisted of 0.1% ammonia in water as solvent A and acetonitrile as solvent B. The flow rate was maintained as 0.4 mL/min throughout the run time of 5 min. Initial composition of solvents were 5:95 A:B, which is retained for 1 min and then the composition was made 10:90 by 2 min and kept up to 2.7 min. The composition was made back to 5:95 by 3.2 min and kept unchanged up to the end.

The levels of curcuminoids and curcumin metabolites were monitored by following the *m*/*z* values of 367.13, 337.12, 307.11, 371.15, 373.16, 375.19, 543.15 and 447.07 for CUR, DMC, BDMC, THC, HHC, OHC, COG and COS respectively, under negative mode.

### 3.3. Preparation of Standard and Quality Control Solutions

A standard stock solution of concentration 500 µg/mL of CUR, DMC, BDMC, COG, and COS were prepared in methanol, the THC, HHC and OHC were prepared in 50:50 methanol: water and all the standard solutions were stored in amber glass bottles with Teflon screw cap at 4 °C. Working standard solutions were prepared so as to obtain the final concentration of 1, 2, 4, 8, 16, 32, 64, 128 and 256 ng/L in 200 µL of blank human plasma for calibration, followed by spiking an aliquot of 10 µL IS (1 µg/mL). The samples were diluted with 100 µL acidified phosphate buffer. 200 µL of ethyl acetate was added to each standard, vortexed and centrifuged. Transferred the supernatant to a fresh glass tube and dried under nitrogen at pressure less than 20 psi at 50 ± 3 °C. Extraction with ethyl acetate was repeated for 2 more times and the dried elute was reconstituted in 200 µL methanol. Then the samples were filtered through 0.2 µM syringe filters and transferred to vials for analysis and injected a volume of 10 µL.

### 3.4. Method Validation Procedures

The calibration standard solutions were employed for establishment of linearity and range. The accuracy and precision were ascertained by the injection of standards at six replicates each in 3 sets on the same day for intra-day repeatability and on six consecutive days for inter-day repeatability.

The intra and inter day accuracy (% bias) of the developed method was determined from mean measured concentrations and nominal concentrations as follows:

(1) $$\;\%\;\mathrm{bias}\;=\left[\frac{\left(\mathrm{mean}\;\mathrm{measured}\;\mathrm{concentration}-\mathrm{nominal}\;\mathrm{concentration}\right)}{\mathrm{nominal}\;\mathrm{concentration}}\right]\;\times 100\;$$

The intra and inter day precision (% relative standard deviation (RSD)) of the developed method was calculated from mean measured concentrations as follows:

(2) $$\;\%\;\mathrm{RSD}=\frac{\mathrm{standard}\;\mathrm{deviation}\;\left(\mathrm{SD}\right)\;\mathrm{of}\;\mathrm{mean}\;\mathrm{measured}\;\mathrm{concentration}}{\mathrm{Mean}\;\mathrm{measured}\;\mathrm{concentration}}\times 100\;$$

### 3.5. Study Design

The bioavailable form of curcuminoids (Cureit™) was used in this study as a curcumin source. Fifteen subjects were received a single dose (500 mg) in the capsule form. The Cureit™ contained 46.5% total curcuminoids (36% CUR, 9.0% DMC and 1.5% BDMC), 43% total carbohydrates, 5% fibers, 2.4% proteins and 3.2% volatile oil which mainly consists of bisacurone, curdione, aromatic turmerone, dihydroturmerone and turmeronol [15]. Fifteen participants were received a single oral dose of 500 mg of Cureit™ in capsule form with 240 mL water for this study and a mouth check was conducted to ensure compliance.

### 3.6. Ethics and Approvals

This study was conducted at Agile Pharma Services, Bangalore, India and the research was carried out in agreement with the clinical research guidelines established by the Drugs and Cosmetics Act, 1940 of India, Drugs and Cosmetics Rules, 1945 of India, Ethical Guidelines for Biomedical Research on Human Participants, 2006 of Indian Council of Medical Research (ICMR) in India, the principles pronounced in the Declaration of Helsinki (Edinburgh, 2000) [28] and the ICH harmonized tripartite guideline regarding Good Clinical Practice (GCP). This study related documents were reviewed by the Independent Ethics Committee (IEC) of Clinicom, Bangalore, India and approved on 25 August 2015. The protocol was registered (CTRI/2016/07/007118) with Clinical Trials Registry India (clinicaltrials.gov).

### 3.7. Inclusion and Exclusion Criteria of Participants

All participants had a body mass index (BMI) of 18.5 to 24.9 kg/m^2^ and no sign of fundamental disease were shown during the pre-study screening, physical examination and medical history and laboratory examinations were executed within 21 days before the beginning of the study. Pre-study screening blood parameters were within normal limits or were considered by the investigator to be of no clinical significance with respect to the participation in this study. All tested participants were negative for hepatitis B and C and were negative or non-reactive for antibodies to HIV 1 and 2.

The exclusion criteria included individuals who were allergic to curcumin or any component of the formulation or some other related drugs and had a history of presence of important disorder. Individuals who exhibited alcohol dependence, alcohol abuse or drug abuse and history of chronic smoking or chronic consumption of tobacco products also were excluded.

Participants were asked about their well-being before check in, prior to dosing on the dosing day and approximately at 2, 4, 6, 12 and 24 h post dose. In addition, during the study, participants were asked to report any side effects spontaneously to the monitoring staff. Dinner was provided to the participants on the pre-study day, and thereafter fasted overnight as well as for four hours after dosing. Water was not allowed before and after one hour of the Cureit™ administration. 

### 3.8. Sample Collection and Preparation

Each pre-dose blood sample (6 mL) was collected within one hour before dosing and a total of 15 blood samples were collected. The post-dose samples (6 mL each) were collected into vacuum tubes containing K_2_EDTA at 0.25, 0.50, 0.75, 1.00, 1.25, 1.50, 1.75, 2.00, 2.25, 2.50, 2.75, 3, 4, 6, 8, 10, 12 and 24 h after dosing. The heparin-lock method was utilized to avoid clotting of blood in the indwelling cannula. Before each blood sample was drawn, 0.5 mL of blood was discarded to prevent the saline diluted blood and heparin from interfering with the analysis. After collection, all the blood samples were stored and transferred to a container pre-cooled with refrigerant gel packs, and subsequently centrifuged at 4000 rpm at 4 °C for 10 min within 60 min of sample collection. Plasmas were separated and 3 mL of each plasma sample was mixed with 10 µL IS solution having a concentration of 10 µg/mL along with phosphate buffer solution (PBS) and then extracted with 3.0 mL of ethyl acetate at room temperature. The upper organic layer was transferred to a glass tube. Two more extractions were done with ethyl acetate, collected and pooled the organic layers and reduced under nitrogen gas. Then 2.0 mL of methanol was used to reconstitute each sample, filtered through a 0.22 µL nylon syringe filter and the samples were analyzed by the validated Q-TOF mass spectrometer method.

### 3.9. Pharmacokinetic and Statistical Analysis

Pharmacokinetic parameters such as extent of absorption (AUC), peak plasma concentration (C_max_), peak time (T_max_), half life (T_½_), clearance (CL), constant of elimination (K_el_) and volume of distribution (vd) were analyzed using the general linear model analysis variance with Origin Pro 8.5. Statistical analyses were executed with a SAS package (SAS Institute Inc., Cary, NC, USA). Values with *p* < 0.05 were considered as statistically significant.

## 4. Conclusions

Three different simple, rapid, specific, accurate and precise UHPLC-QTOF-MS methods were developed and validated for simultaneous quantification of curcuminoids and CUR metabolites in human blood plasma. To date, there is no validated, reproducible method available for quantification of HHC and OHC from blood plasma. The protocols described in this study provide the comprehensive analytic methods to characterize and quantify the curcuminoids and their metabolites in human plasma after the oral ingestion of CUR as well as different CUR formulations. In this study, the bioavailability of curcuminoids was assessed along with a detailed pharmacokinetics of its metabolites like THC, HHC, OHC, COG and COS in Cureit™, which is curcumin inside a complete turmeric matrix. It is clear that the synergism of curcumin with other bioactive molecules of turmeric made a positive impact in generating high concentration of “free curcuminoids” in the blood plasma as seen in the pharmacokinetic data. The concentration of bioactive metabolites like THC, HHC, OHC, COG and COS were also high in the blood plasm when using Cureit™.

## Figures and Tables

**Table 1:** The peak intensity of the curcuminoids and curcumin metabolites during direct infusion under positive and negative modes

Standards	Intensity I_max_ (×10^3^ cps)	
	Positive Mode	Negative Mode
CUR	10.8	42
DMC	12.3	57.5
BDMC	11.2	63.03
THC	7.9	43
HHC	5.1	22.88
OHC	0.9	3.52
COG	2.8	7.195
COS	0.18	1.895

**Table 2:** Intra and inter day assay accuracy (% bias) data for CUR, DMC, BDMC, THC, HHC, OHC, COG and COS.

Compound	Nominal Conc. (ng/mL)	Intra-Assay			Inter-Assay		
		Set 1	Set 2	Set 3	Set 1	Set 2	Set 3
CUR	4	7.125	6.292	9.667	6.108	7.067	2.817
	8	3.592	13.950	−0.379	7.317	7.379	−2.979
	16	1.385	0.577	−2.098	1.288	2.415	2.354
	32	0.487	1.851	2.794	2.268	3.550	0.873
	64	−3.866	1.203	−1.182	1.620	−0.235	1.046
	128	1.329	0.039	−1.309	−0.840	0.306	−0.203
	256	0.313	0.384	0.126	−0.780	0.193	−0.212
DMC	4	2.842	6.200	−2.992	5.667	0.758	5.525
	8	1.100	5.425	−7.587	3.075	3.783	1.492
	16	2.035	2.271	−0.896	2.023	1.448	3.040
	32	−0.196	2.447	0.860	0.841	1.664	0.080
	64	1.882	−0.136	−1.713	0.634	0.556	0.948
	128	−0.260	0.258	−0.088	−0.126	0.043	0.210
	256	−0.008	−0.086	0.271	0.143	0.333	−0.760
BDMC	4	0.633	2.392	3.108	4.192	0.692	1.767
	8	4.317	2.508	−2.871	7.167	4.854	0.296
	16	0.208	3.646	2.667	4.337	2.167	2.356
	32	−0.632	0.948	0.342	1.740	−0.793	2.111
	64	−5.248	0.324	1.055	0.106	0.451	−0.172
	128	0.779	−0.310	−0.105	0.367	−0.182	0.454
	256	0.206	−0.197	0.770	0.560	−0.509	0.444
THC	4	1.483	0.075	−2.358	−3.333	2.800	−2.358
	8	15.000	0.908	5.783	2.300	−5.908	−1.712
	16	4.375	4.583	5.823	4.104	1.652	3.748
	32	−1.563	−2.708	2.653	2.365	1.337	2.627
	64	−0.573	0.365	−0.009	−0.157	0.038	0.460
	128	−2.552	−1.250	−1.172	−0.422	−0.414	0.578
	256	0.547	−1.094	−0.394	0.165	−0.173	0.642
HHC	4	0.658	8.175	0.925	3.250	3.600	0.450
	8	−10.000	−4.667	0.621	3.971	−5.458	−0.650
	16	−5.208	−2.083	4.517	1.106	−0.527	−0.215
	32	3.125	1.354	2.694	3.232	0.439	−2.377
	64	0.990	0.469	2.398	1.955	1.144	0.789
	128	0.990	−1.120	0.010	0.024	−0.421	0.499
	256	−0.352	−1.328	0.085	−0.304	0.123	−0.096
OHC	4	2.808	9.908	7.494	4.883	6.550	−0.042
	8	1.208	0.300	1.883	−2.842	3.133	0.192
	16	−3.448	3.789	−0.919	1.071	2.378	−2.581
	32	11.098	−3.083	−0.706	−1.488	1.964	4.443
	64	−1.296	−0.001	−0.800	1.420	0.310	0.252
	128	−0.487	1.633	0.733	0.438	−0.558	−0.582
	256	2.931	0.624	−0.092	0.774	0.119	−0.122
COG	4	8.833	5.833	2.833	1.500	0.083	−1.750
	8	11.583	10.458	9.375	7.333	5.375	3.708
	16	2.771	1.708	1.208	0.604	0.292	−0.542
	32	0.354	−0.052	0.177	0.021	−0.125	−0.354
	64	1.620	1.417	1.448	0.903	0.391	0.469
	128	0.891	0.646	0.143	−0.339	−0.982	−1.070
	256	0.807	0.440	0.064	0.135	−0.089	−0.010
COS	4	12.750	6.750	4.667	1.750	1.250	0.667
	8	3.692	1.958	1.250	0.792	0.000	−1.000
	16	0.479	0.437	0.081	−0.375	−0.648	−0.604
	32	1.010	0.531	0.156	−0.229	−0.094	−0.260
	64	0.104	−1.615	−0.260	−0.260	−0.245	−0.391
	128	1.510	0.208	0.122	0.065	−0.008	−0.388
	256	0.833	0.612	0.208	0.273	−0.508	0.013

**Table 3:** Intra and inter day assay precision (% RSD) data for CUR, DMC, BDMC, THC, HHC, OHC, COG and COS.

Compound	Nominal Conc. (ng/mL)	Intra-Assay			Inter-Assay		
		Set 1	Set 2	Set 3	Set 1	Set 2	Set 3
CUR	4	7.262	8.894	9.779	7.997	7.654	10.411
	8	5.277	6.242	10.188	9.517	7.312	3.263
	16	2.345	1.218	3.167	5.532	6.090	3.969
	32	1.233	1.895	3.207	2.714	2.196	4.052
	64	8.293	4.409	2.992	1.989	3.654	2.239
	128	2.096	1.486	2.736	3.046	1.734	3.258
	256	1.071	2.535	1.916	2.151	1.015	1.348
DMC	4	5.398	11.478	3.098	12.374	3.157	7.751
	8	6.095	11.009	9.751	4.730	8.282	2.955
	16	3.984	2.158	3.287	8.958	7.664	7.691
	32	0.655	3.251	5.937	3.342	1.759	4.120
	64	2.741	0.716	3.144	1.219	2.473	3.351
	128	0.972	1.363	2.816	1.369	1.503	1.662
	256	1.502	1.277	1.620	1.724	1.347	0.913
BDMC	4	5.938	11.762	10.882	15.058	7.284	10.178
	8	1.370	3.183	9.644	5.040	8.077	8.446
	16	2.632	7.026	6.027	3.710	4.717	2.280
	32	1.662	2.784	5.402	7.436	6.178	2.302
	64	9.533	3.070	4.464	1.186	2.386	1.088
	128	0.467	1.974	1.326	0.001	2.390	1.888
	256	0.554	1.436	0.913	0.753	0.548	0.401
THC	4	5.579	6.357	13.441	7.371	3.823	7.225
	8	3.919	5.693	7.311	9.320	7.633	8.456
	16	3.111	1.826	2.045	1.808	1.775	4.042
	32	4.444	1.617	1.012	0.678	1.262	3.167
	64	0.595	0.589	1.081	0.961	0.265	2.626
	128	2.567	2.487	1.856	1.745	0.813	1.904
	256	0.441	2.159	1.314	1.200	0.591	2.399
HHC	4	8.815	8.591	12.896	10.771	9.761	5.472
	8	3.675	4.065	8.366	4.387	8.154	7.690
	16	4.390	1.950	4.280	5.162	5.346	4.706
	32	2.922	1.780	2.539	1.988	3.101	3.041
	64	2.572	1.794	2.428	1.536	2.259	0.871
	128	0.473	1.767	1.153	0.337	0.732	1.740
	256	2.310	1.758	0.785	1.693	0.742	1.641
OHC	4	5.745	6.905	5.793	10.637	7.220	15.026
	8	11.047	4.623	5.516	4.263	5.420	9.732
	16	4.897	0.934	1.574	3.270	4.085	3.412
	32	6.194	2.930	2.471	3.221	2.910	4.476
	64	1.418	1.615	0.999	0.849	2.548	0.492
	128	3.845	2.266	0.866	2.590	1.290	0.641
	256	3.335	0.311	0.488	0.108	0.572	0.672
COG	4	1.036	2.548	1.857	0.739	0.577	0.917
	8	0.342	0.643	0.229	1.195	1.438	1.053
	16	0.274	0.284	0.560	0.404	0.415	0.837
	32	0.140	0.260	0.126	0.126	0.217	0.048
	64	0.988	0.447	0.170	0.580	0.322	0.823
	128	0.046	0.126	0.863	0.623	0.229	0.705
	256	0.334	0.089	0.352	0.096	0.242	0.022
COS	4	2.614	1.021	0.365	1.277	1.132	1.831
	8	0.430	1.691	1.111	0.559	0.250	1.456
	16	0.423	0.311	0.694	0.664	1.137	0.729
	32	0.449	0.824	0.437	0.665	0.325	0.231
	64	0.238	1.441	0.326	0.261	0.182	0.656
	128	0.740	0.238	0.035	0.016	0.028	0.327
	256	0.667	0.594	0.592	0.281	0.416	0.045

**Table 4:** Pharmacokinetic parameters of CUR, DMC, BDMC, COG, COS, THC, HHC and OHC after oral administration of a single dose of Cureit™ (500 mg/day).

Compounds	AUC (ng·h·mL^−1^)	C_max_ (ng·mL^−1^)	T_max_ (h)	T_½_ (h)	CL (10^6^ h^−1^·mL^−1^)	K_el_ (h^−1^)	Vd (10^6^ mL^−1^)
CUR	527.100	74.310	4.000	1.681	0.949	0.412	2.300
DMC	147.000	17.193	4.000	1.569	3.401	0.442	7.699
BDMC	72.610	8.280	4.000	1.480	6.886	0.468	14.710
COG	3.200	0.862	2.500	0.301	156.250	2.303	67.846
COS	0.900	1.060	2.250	0.300	555.560	2.309	240.580
THC	419.700	42.840	12.000	4.673	1.191	0.148	8.033
HHC	121.300	10.330	12.000	11.574	4.122	0.060	68.840
OHC	41.510	4.910	12.000	2.768	12.045	0.250	48.116

## References

[1] Cao Y., Xu R.X., Liu Z. (2014). A high-throughput quantification method of curcuminoids and curcumin metabolites in human plasma via high-performance liquid chromatography/tandem mass spectrometry. J. Chromatogr. B.

[2] Huang Y.-S., Hsieh T.-J., Lu C.-Y. (2015). Simple analytical strategy for MALDI-TOF-MS and nanoUPLC–MS/MS: Quantitating curcumin in food condiments and dietary supplements and screening of acrylamide-induced ROS protein indicators reduced by curcumin. Food Chem..

[3] Anand P., Sundaram C., Jhurani S., Kunnumakkara A.B., Aggarwal B.B. (2008). Curcumin and cancer: An “old-age” disease with an “age-old” solution. Cancer Lett..

[4] Amalraj A., Pius A., Gopi S., Gopi S. (2017). Biological activities of curcuminoids, other biomolecules from turmeric and their derivatives—A review. J. Tradit. Complement. Med..

[5] Dhillon N., Aggarwal B.B., Newman R.A., Wolff R.A., Kunnumakkara A.B., Abbruzzese J.L., Ng C.S., Badmaev V., Kurzrock R. (2008). Phase II trial of curcumin in patients with advanced pancreatic cancer. Clin. Cancer Res..

[6] Anand P., Kunnumakkara A.B., Newman R.A., Aggarwal B.B. (2007). Bioavailability of curcumin: Problems and promises. Mol. Pharm..

[7] Marczylo T.H., Steward W.P., Gescher A.J. (2009). Rapid analysis of curcumin and curcumin metabolites in rat biomatrices using a novel Ultraperformance Liquid Chromatography (UPLC) method. J. Agric. Food Chem..

[8] Dempe J.S., Scheerle R.K., Pfeiffer E., Metzler M. (2013). Metabolism and permeability of curcumin in cultured Caco-2 cells. Mol. Nutr. Food Res..

[9] Motomu S., Kiyotaka N., Akio W., Tsuyoshi T., Teiko Y., Shigefumi K., Fumiko K., Teruo M. (2014). Comparison of the effects of curcumin and curcumin glucuronidein human hepatocellular carcinoma HepG2 cells. Food Chem..

[10] Lou Y., Zheng J., Hu H., Lee J., Zeng S. (2015). Application of ultra-performance liquid chromatography coupled with quadrupole time-of-flight mass spectrometry to identify curcumin metabolites produced by human intestinal bacteria. J. Chromatogr. B.

[11] Asai A., Miyazawa T. (2000). Occurrence of orally administered curcuminoid as glucuronide and glucuronide/sulfate conjugates in rat plasma. Life Sci..

[12] Ireson C.R., Orr S., Jones D.J.L., Verschoyle R., Lim C.K., Luo J.L., Howells L., Plummer S.M., Jukes R.M., Williams Steward W.P. (2001). Characterization of metabolites of the chemopreventive agent curcumin in humans and rat hepatocytes and in rat plasma and evaluation of their ability to inhibit phorbol ester-induced prostaglandin E2 production. Cancer Res..

[13] Gopi S., Jacob J., Mathur K.Y. (2016). Acute and subchronic oral toxicity studies of hydrogenated curcuminoid formulation ‘CuroWhite’ in rats. Toxicol. Rep..

[14] Ireson C.R., Jones D.J., Orr S., Coughtrie M.W., Boocock D.J., Williams M.L., Farmer P.B., Steward W.P., Gescher A.J. (2002). Metabolism of the cancer chemopreventive agent curcumin in human and rat intestine. Cancer Epidemiol. Biomark. Prev..

[15] Amalraj A., Jude S., Varma K., Jacob J., Gopi S., Oluwafemi O.S., Thomas S. (2017). Preparation of a novel bioavailable curcuminoid formulation (Cureit™) using Polar-Nonpolar-Sandwich (PNS) technology and its characterization and applications. Mater. Sci. Eng. C.

[16] Gopi S., George R., Jude S., Sriraam V.T. (2014). Cell culture study on the cytotoxic effects of “Cureit”—A novel bio available curcumin-anti cancer effects. J. Chem. Pharm. Res..

[17] Gopi S., George R., Sriraam V.T. (2014). Cell culture study on the effect of bioavailable curcumin—“Cureit” on elastase inhibition activity. Br. Biomed. Bull..

[18] Gopi S., George R., Sriraam V.T. (2014). Antioxidant potential of “Cureit”—A novel bioavailable curcumin formulation. Asian J. Pharm. Tech. Innov..

[19] Gopi S., George R., Sriraam V.T. (2014). Cell culture study on the effects of “Cureit” hyaluronidase inhibition—Anti aging effects. Int. J. Curr. Res..

[20] Gopi S., George R., Thomas M., Jude S. (2015). A pilot cross-over study to assess the human bioavailability of “Cureit”—A bioavailable curcumin in complete natural matrix. Asian J. Pharm. Tech. Innov..

[21] Ahmad N., Warsi M.H., Iqbal Z., Samim M., Ahmad F.J. (2014). Quantification of curcumin, demethoxycurcumin, and bisdemethoxycurcumin in rodent brain by UHPLC/ESI-Q-TOF-MS/MS after intra-nasal administration of curcuminoids loaded PNIPAM nanoparticles. Drug Test. Anal..

[22] Lin J.K., Pan M.H., Lin-Shiau S.Y. (2000). Recent studies on the biofunctions and biotransformations of curcumin. Biofactors.

[23] Zhongfa L., Chiu M., Wang J., Chen W., Yen W., Fan-Havard P., Yee L.D., Chan K.K. (2012). Enhancement of curcumin oral absorption and pharmacokinetics of curcuminoids and curcumin metabolites in mice. Cancer Chemother. Pharmacol..

[24] Vareed S.K., Kakarala M., Ruffin M.T., Crowell J.A., Normolle D.P., Djuric Z., Brenner D.E. (2008). Pharmacokinetics of curcumin conjugate metabolites in healthy human subjects. Cancer Epidemiol. Biomarkers Prev..

[25] Liu Z., Xie Z., Jones W., Pavlovicz R.E., Liu S., Yu J., Li P.K., Lin J., Fuchs J.R., Marcucci G. (2009). Curcumin is a potent DNA hypomethylation agent. Bioorg. Med. Chem. Lett..

[26] Gopi S., Jacob J., Varma K., Jude S., Amalraj A., Arundhathy C.A., George R., Sreeraj T.R., Divya C., Kunnumakkara A.B. (2017). Comparative Oral Absorption of Curcumin in a Natural Turmeric Matrix with Two Other Curcumin Formulations: An Open-label Parallelarm Study. Phytother. Res..

[27] Verma M.K., Najar I.A., Tikoo M.K., Singh G., Gupta D.K., Anand R., Khajuria R.K., Sharma S.C., Johri R.K. (2013). Development of a validated UPLC-qTOF-MS Method for the determination of curcuminoids and their pharmacokinetic study in mice. Daru. J. Pharm. Sci..

[28] Carlson R.V., Boyd K.M., Webb D.J. (2004). The revision of the Declaration of Helsinki: Past, present and future. Br. J. Clin. Pharmacol..

